# Opposites Attract: Assortative Mating and Immigrant–Native Intermarriage in Contemporary Sweden

**DOI:** 10.1007/s10680-019-09546-9

**Published:** 2019-12-11

**Authors:** Annika Elwert

**Affiliations:** Department of Economic History, Centre for Economic Demography, Lund University, Alfa 1, Scheelevägen 15B, Box 7083, 220 07 Lund, Sweden

**Keywords:** Intermarriage, Assortative mating, Age homogamy, Status exchange, Sweden

## Abstract

This paper studies how immigrant–native intermarriages in Sweden are associated with individual characteristics of native men and women and patterns of assortative mating. Patterns of educational- and age-assortative mating that are similar to those found in native–native marriages may reflect openness to immigrant groups, whereas assortative mating patterns that indicate status considerations suggest that country of birth continues to serve as a boundary in the native marriage market. The study uses Swedish register data that cover the entire Swedish population for the period of 1991–2009. The results from binomial and multinomial logistic regressions show that low status of natives in terms of economic and demographic characteristics is associated with intermarriage and that intermarriages are characterized by educational and age heterogamy more than are native–native marriages. The findings indicate that immigrant women as well as immigrant men become more attractive marriage partners if they are considerably younger than their native spouses. This is particularly true for intermarriages with immigrants from certain regions of origin, such as wives from Asia and Africa and husbands from Asia, Africa, and the Middle East. Gender differences in the intermarriage patterns of native men and women are surprisingly small.

## Introduction

A distinct feature of many marriage markets is homogamy in spousal choice. Partners tend to be similar with regard to socioeconomic status (Kalmijn 1991), age (van Poppel et al. 2001), education, race, and religion (Blackwell and Lichter 2004). While there was increasing similarity in certain characteristics such as education and age over several decades (Schwartz and Mare 2005; Van de Putte et al. 2009), there was a decrease in homogamy in terms of country of birth during the rise of intermarriages between natives and immigrants in Europe.^1^ Scholars often study immigrant–native intermarriage in the context of immigrant integration and regularly regard intermarriage as the final step in the assimilation process (Gordon 1964). A rather neglected aspect of this is that “it takes two to tango”: it requires as much willingness on the part of natives to intermarry as it does on the part of immigrants. This study addresses a topic that has hitherto been understudied in that it analyses the (inter-)marriage behaviour of native Swedes.^2^ Focusing on the native majority expands the intermarriage literature and leads to a better understanding of societal openness towards minorities in the majority’s marriage market. By taking into account the characteristics of both the native partner and the immigrant partner, this paper is an important contribution to the intermarriage literature. Intermarriage is often thought to signal the fact that different social groups regard one another as equals (cf. Kalmijn 1991), but marriage can also reproduce social hierarchies by excluding certain groups from the pool of potential partners and reproducing social structures within these. Where intermarriages display systematic patterns of hypergamy and hypogamy, that is, native partners marry up or down in characteristics such as age and education, it can be concluded that the partners do not regard each other as social equals (Merton 1941). Intermarriage patterns therefore have the potential to reveal implicit hierarchies of immigrants in the marriage market.^3^

The particular question that this paper attempts to answer is whether intermarriages are associated with the status of native Swedes as well as that of immigrants in the Swedish marriage market. By analysing the *individual characteristics of natives* that are associated with intermarriage as well as the *educational- and age*-*assortative mating patterns* of intermarried couples rather than the mere frequency of such unions, this study contributes to a previously understudied area in the intermarriage literature. It uses high-quality register data covering the entire population of residents in Sweden and includes all marriages and non-marital unions with common children that were established in the period 1991–2009.

## Background and Previous Research on Immigrant–Native Intermarriages in Europe and Sweden

**Fig. 1:**
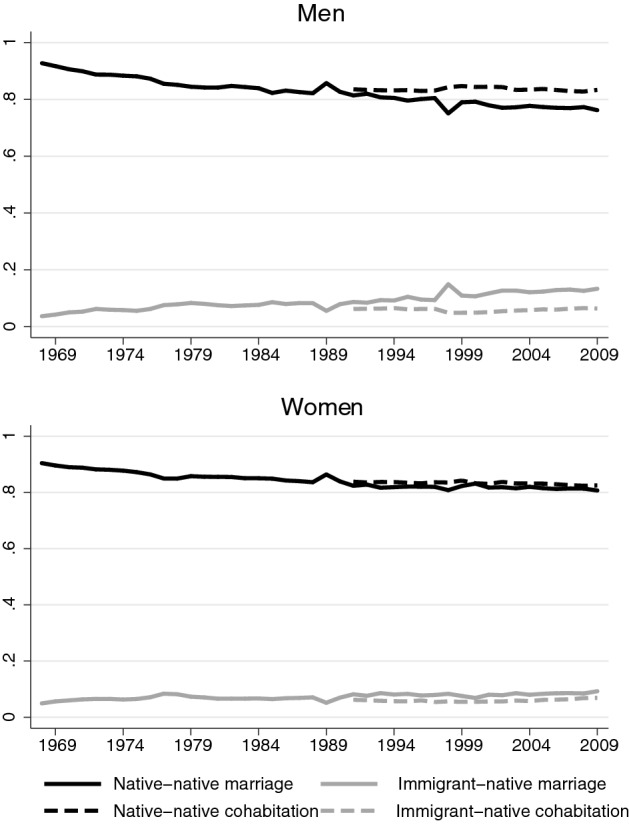
Shares of native–native unions and immigrant–native unions of all unions of native Swedish men and women in Sweden 1969–2009

Intermarriage between immigrants and natives has increased in most European countries in past decades and is closely related to the proportion of immigrants in the country (Lanzieri 2012). This general increase in intermarriage in Europe is largely related to a substantial increase in intermarriage with spouses from countries outside the EU (de Valk and Medrano 2014). Intermarriage rates in Sweden have risen continuously since the 1970s, and the increase is somewhat steeper for men than for women. Figure 1 displays the proportions of immigrant–native intermarriages (defined here as marriages between a native Swede^4^ and their foreign-born spouse) and native–native marriages (defined as marriages between two native spouses) of all newly contracted marriages made by native Swedes from 1969 to 2009. As of 1991 the register extracts used in this paper contain an identifier for non-marital cohabitations with common children, which makes it possible to report the shares of native–native cohabitation and immigrant–native cohabitation.

For native women, the shares of immigrant–native marriage and cohabitation are close in size and have changed only marginally since the 1990s; for native men, there is a wider gap with immigrant–native cohabitation displaying lower rates with little increase over time and immigrant–native marriages displaying higher rates with a more pronounced increase over time.

In earlier decades, intermarriage between native Swedes and immigrants was dominated by intermarriage with other Nordic citizens, particularly Finns (Cretser 1999). In more recent years, the increase in intermarriages can be largely accounted for by the increased number of marriages with partners from outside Europe, and Thailand has replaced Finland as the most frequent country of origin for intermarried immigrant women (although Finland remains the most common country of origin for intermarried immigrant men; Haandrikman 2014).

Most of the previous research on intermarriage has a strong emphasis on immigrant integration (Kulu and González-Ferrer 2014). Intermarriage in this regard is thought to indicate social integration (Kalmijn 1998). Studies in this line of research largely focus on the intermarriage *rates* of different immigrant groups, since these are understood to be a measure of group closure and social distance. Individual characteristics that are associated with immigrant–native intermarriage are typically contrasted with those that are associated with endogamous immigrant marriage (for example, González-Ferrer 2006; Kalmijn and van Tubergen 2006; Dribe and Lundh 2008 on Sweden). Studies that take into account the native side of intermarriage are comparatively rare. They often show that native men who intermarry are more often lower educated and have a lower income (Guetto and Azzolini 2015; Haandrikman 2014), are older (Glowsky 2007; Guetto and Azzolini 2015; Niedomysl et al. 2010), and may encounter difficulties in finding an attractive partner in the native endogamous marriage market—difficulties that could be related to their age (Glowsky 2007) or to local shortages of native women (Östh et al. 2011).

As Kulu and González-Ferrer (2014) note, patterns of intermarriages are gendered. In many countries, more foreign-born women than foreign-born men are intermarried (and the reverse applies to natives). Such patterns can be explained by selective immigration and the structural constraints of the marriage market or by selective marriage patterns of the native majority population. In contrast to other European countries, these patterns in Sweden’s case can be partly explained by gendered patterns of *marriage migration;* i.e. native men (and to a lesser extent native women) marrying women (men) from economically poorer countries. Marriage migration to the native majority is a growing phenomenon particularly in Sweden, although it does exist elsewhere as well (de Valk and Medrano 2014). Niedomysl et al. (2010) show that assortative mating patterns in marriages of marriage migrants and natives deviate from the Swedish norm. While both the opportunity structures and individual characteristics may be related to the likelihood of marrying a marriage migrant, individual characteristics such as age and income appear to be of greater importance (Östh et al. 2011).

## Theory and Hypotheses

The prevalence of intermarriages in a society, although dependent on opportunity structures, is often viewed as a signal of the successful integration of immigrants (Gordon 1964; see Rodríguez-García 2015 for an overview). However, as much as intermarriage reflects immigrants’ partner preferences, it also reflects those of the native majority.

### Hierarchies of Immigrants in the Native Partner Market

It is impossible to determine from marriage rates alone if immigrant or native majority preferences have a larger impact in setting boundaries between immigrants and natives in the partner market. Studies that look beyond intermarriage rates and focus on the native majority’s preferences by analysing online dating data have consistently shown the existence of ethnic and racial hierarchies in the majority’s dating markets (for example, Jakobsson and Lindholm 2014 for Sweden; Lin and Lundquist 2013 for the USA; Potârcă and Mills 2015 for Europe). Studies on ethnic preferences in the Swedish partner market consistently show that in Sweden, as in many other European countries, Europeans rank at the top, followed by Hispanic, Asian, African and Arab individuals, the latter being at the bottom of the hierarchy (Potârcă and Mills 2015; Jakobsson and Lindholm 2014). Moreover, white Europeans (in Sweden) perceive a hierarchy within the group of European countries whereby Sweden ranks at the top, followed by other Scandinavians, Western Europeans, Southern Europeans, and finally Eastern Europeans who are ranked close to non-European groups such as Latin Americans (Osanami Törngren 2016).

Intermarriage rates between immigrants and natives reflect only the frequency of intermarriage, not its particular nature. The question whether intermarriage reflects societal openness or a hierarchy of immigrants cannot be resolved without also accounting for patterns of assortative mating by characteristics such as age and education within these unions. They have the potential to reveal something about the nature of these unions; namely, where the conditions under which members of the native majority accept someone as a marriage partner differ between immigrants and natives (Alba and Foner 2015). Assortative mating patterns in intermarriages can be either the result of more openness and less pronounced homogamy preferences or an indication of status considerations, when only immigrants with certain traits are considered marriageable.

Besides immigrants’ characteristics, immigrants’ residence status may determine their status in the partner market. Some studies have suggested that there is potentially a hierarchy among migrants who have secured residence in the host country versus (prospective) immigrants without secured residence or who lack the option of a legal entry to the destination country. For example, a study on Italy found evidence for the importance of citizenship in marriage behaviour (Azzolini and Guetto 2017). I assume similar mechanisms to be at work for migrants who migrate for the purpose of marriage. Marriage migration to Western countries is a noticeable yet rarely quantified phenomenon (Beck and Beck-Gernsheim 2014; see Niedomysl, Östh and van Ham 2010 for a first attempt to quantify the scope of marriage migration to Sweden). Because marriage migration to natives falls under the legal framework of family migration (Kofman 2004), marriage or other forms of conjugal union with a Swedish partner (which, legally speaking, are largely the same in Sweden) give non-residents the opportunity to obtain a residence permit. Under the conditions of restricted entry into Western countries, marriage migration is one of few opportunities to obtain a residence permit. Thus, marriage migrants constitute a particular group of migrants in the Swedish partner market, and the marriage patterns of these marriages can differ from both native endogamous marriages and intermarriages with immigrants who are long-term residents of the country.

### The Openness Hypothesis

Patterns of marital homogamy and heterogamy in general (Kalmijn 1991) and intermarriage patterns in particular (Elwert 2018) reflect social stratification and openness in the society. On the individual level, marriage implies that spouses regard each other as partners for life on equal terms. Assortative mating patterns can thus reveal the relative importance of homogamy with respect to different characteristics. The highly educated, for example, may have strong preferences for educational homogamy (Schwartz 2013) but may be more open towards inter-ethnic marriage (Hello et al. 2002; Wagner and Zick 1995). With respect to assortative mating patterns in immigrant–native intermarriages by, for example, age and education, openness would mean that assortative mating patterns found in intermarriages do not differ from those found in native endogamous marriages. Such pattern would indicate lower homogamy preferences with regard to partner origin but still high homogamy preferences in other respects such as education and age. Alternatively, intermarriages could be an indication of a generally lower level of homogamy preferences and more openness towards other social groups (such as educational groups and social classes). From the *openness* perspective, immigrant partners are accepted as equal partners for life, and the patterns of assortative mating in these marriages on characteristics such as education and age do not *systematically* differ from those in native endogamous marriages. That means that these unions are (1) similar to native endogamous unions in terms of age and educational homogamy, or (2) are more heterogamous than native endogamous unions, that is, natives more often marry up (hypergamy) *and* down (hypogamy) in these characteristics.

The Openness Hypothesis (H1): Assortative mating patterns in intermarriages and endogamous Swedish unions do not differ systematically.

If higher odds of intermarriage are related to openness towards immigrants, no differences between migrant groups and by gender should be observed.^5^ For an overview of the hypotheses and their different meaning in native men–immigrant women and native women–immigrant men unions as well as in unions with resident migrants compared to unions with marriage migrants, see Table 1.

**Table 1:** The hypotheses by gender and migrant type

	H1 The openness hypothesis
General hypothesis	Assortative mating patterns in intermarriages and endogamous Swedish unions do not differ systematically. They are not significantly different to native endogamous unions. Alternatively, they show higher levels of heterogamy in the form of higher hypergamy and higher hypogamy
Unions of native men and immigrant women	Assortative mating patterns in unions of native men and immigrant women are not significantly different to native endogamous unions. Alternatively, they show higher levels of heterogamy in the form of higher hypergamy and higher hypogamy
Unions of native women and immigrant men	Assortative mating patterns in unions of native women and immigrant men are not significantly different to native endogamous unions. Alternatively, they show higher levels of heterogamy in the form of higher hypergamy and higher hypogamy
Unions with resident migrants versus unions with marriage migrants	Assortative mating patterns in unions with resident migrants and unions with marriage migrants do not differ systematically. They are not significantly different to native endogamous unions. Alternatively, they show higher levels of heterogamy in the form of higher hypergamy and higher hypogamy

	H2 The attractiveness hypothesis
General hypothesis	Natives’ lower status in economic (lower education, lower income) and demographic characteristics (being older, having had previous relationships) increases the odds of marrying immigrants, particularly immigrants of lower status
Unions of native men and immigrant women	Native men who have lower status in economic characteristics (lower education, lower income) and demographic characteristics (being older, having had previous relationships) have higher odds of marrying immigrant women, particularly immigrant women of lower status
Unions of native women and immigrant men	Native women who have lower status in economic characteristics (lower education, lower income) and demographic characteristics (being older, having had previous relationships) have higher odds of marrying immigrant men, particularly immigrant men of lower status
Unions with resident migrants versus unions with marriage migrants	Lower status in economic (lower education, lower income) and demographic characteristics (being older, having had previous relationships) increases the odds of marrying marriage migrants compared to marrying immigrants with prior residence in Sweden

	H3 The status exchange hypothesis
General hypothesis	The lower the immigrant group’s status is in the marriage market, the more likely is that status exchange patterns are observed
Unions of native men and immigrant women	The lower the immigrant group’s status is in the marriage market, the more likely status exchange patterns take the form of age hypogamy for native men
Unions of native women and immigrant men	The lower the immigrant group’s status is in the marriage market, the more likely status exchange patterns take the form of educational hypergamy for native women
Unions with resident migrants versus unions with marriage migrants	Status exchange in the form of natives’ educational hypergamy and age hypogamy is more pronounced in marriage migrant marriages than in marriages with immigrants with prior residence in Sweden

### The Attractiveness Hypothesis

Under the condition of a hierarchy of immigrants in the marriage market, immigrants with low status are considered less attractive marriage partners than natives (or immigrants with high status). Following this argument, even frequent immigrant–native intermarriages do not necessarily reflect the fact that individuals who intermarry are more open and have lower homogamy preferences. Analysing the individual characteristics of natives that are associated with marrying immigrants as well as assortative mating patterns in these unions is thus more important than the mere frequency of intermarriage for drawing conclusions about natives’ openness towards immigrants as marriage partners. Other than a sign of openness, intermarriages can also be a result of competition for high-status individuals (Gullickson and Torche 2014; Kalmijn 1994). If there is competition in the marriage market for high-status partners, high-status individuals will end up choosing one another and leave low-status individuals to marry each other. Low status in the native endogamous marriage market is related to characteristics such as education and income, but can also be related to non-economic characteristics such as age (older individuals), physical attractiveness, being divorced or having children from previous relationships. Status in the marriage market can be different for men and women, particularly in less gender-equal societies (Kalmijn 1994): women compete for spouses with attractive economic resources, while men compete for spouses who are attractive in terms of other characteristics such as physical attractiveness or lower age.

According to the *attractiveness* perspective, economic and non-economic individual characteristics are important status markers that signal attractiveness in the native marriage market. Education and income signal a person’s attractiveness in economic characteristics. Age and the number of previous committed relationships (that is, being divorced or having children from previous non-marital unions) are demographic characteristics, which affect a person’s status in the marriage market. Natives who have low status in the marriage market may then be pushed into marrying other low-status individuals, namely immigrants.^6^ The attractiveness perspective relates to individual characteristics of native men and women and not to assortative mating patterns in intermarriages. It states that lower individual attractiveness increases the odds of being in a union with an immigrant.

The Attractiveness Hypothesis (H2): Natives’ lower status in economic (lower education, lower income) and demographic characteristics (being older, having had previous relationships) increases the odds of being in a union with an immigrant.

Such patterns may be observed in particular among intermarriages with immigrants of lower status or who have less secured residence (marriage migrants), who are likely to be regarded the least attractive partners in the marriage market. I report group-specific hypotheses in Table 1.

### The Status Exchange Hypothesis

The status exchange hypothesis, which originates from the works of Davis (1941) and Merton (1941), is closely related to the notions of attractiveness in the marriage market. Status exchange focuses on the joint characteristics of couples (assortative mating) rather than how the partners’ individual characteristics are associated with intermarriage. Davis (1941) and Merton (1941) hypothesized that under the conditions of an ethnic hierarchy in the marriage market whereby individuals with low ethnic status are regarded as less attractive partners, ethnic boundaries are only crossed if the ethnic minority partner is otherwise an attractive marriage partner (Fu 2001). The key notion is that the partner with low ethnic status compensates the partner with high ethnic status (the native) by offering high status in terms of other characteristics. A good example of the trading of class status for racial status is one in which less educated white females marry highly educated black males (Qian 1997; Gullickson 2006; see Gullickson and Torche 2014 for status exchange with more racial categories).

In the conventional formulation of the status exchange hypothesis, status exchange patterns are mostly found in unions between highly educated black males and low educated white females (Gullickson 2006; Hou and Myles 2013), reflecting traditional patterns of female educational hypergamy.^7^ Despite an increased prevalence of female educational hypogamy in Sweden (Esteve et al. 2016; Chudnovskaya 2016), native Swedish women still report stronger preferences for partners with higher resource levels than do men (Gustavsson et al. 2008).

In the traditional formulation of the status exchange hypothesis, scholars typically think of status as a higher social class or level of education (Kalmijn 1993). Marriage market status is, however, more multidimensional than that: several studies have shown that status in characteristics other than economic ones, such as general physical attractiveness (Taylor and Glenn 1976), body mass (Chiappori et al. 2012), and inherited family prestige (Almenberg and Dreber 2009), can be exchanged for economic status. Therefore, a broader perception of the status exchange hypothesis can also include an exchange of status in other characteristics for immigrant status (as stated in Hypothesis 3, which I explain for the examples of age below).

The Status Exchange Hypothesis (H3): The lower the immigrant group’s status is in the marriage market, the more likely is that status exchange patterns are observed.

Various studies based on online dating data have reported preferences for younger partners (Alterovitz and Mendelsohn 2011; Hitsch et al. 2010; Rudder 2014; Skopek et al. 2011). These studies consistently show that women tend to prefer men of approximately their own age, while men prefer somewhat younger women and rate women in their early twenties as being the most attractive, independent of their own age. Only once they are in their forties do women begin to rate men younger than themselves as being the most attractive (Rudder 2014). Despite this type of data not being representative of society as a whole, it nevertheless shows clear evidence of younger age being a proxy for physical attractiveness, particularly from the male perspective. Moreover, female age hypergamy, i.e. women marrying older men, shows a remarkably stable pattern in many countries (e.g. Esteve et al. 2009; Kolk 2015 for Sweden) and may be related to a representation of male superiority (Bourdieu 2002). Thus, age and specifically the *age gap between spouses* are likely to be an asset for status exchange.^8^

Taking these gendered patterns of partner preferences into account, it is likely that native women seek highly educated immigrant partners and that native men seek immigrant partners who are younger (see Table 1 for the gender-specific hypotheses). These (gendered) patterns of status exchange are likely to be more pronounced in marriages with marriage migrants than in marriages with resident immigrants. The status that native Swedes have to offer in unions with marriage migrants is not merely a higher status based on belonging to the majority but potentially a legal entry to the country and a residence permit.

## Data and Method

The analysis is based on register data maintained by Statistics Sweden. The sample created for this study contains all marriages and non-marital cohabitations with common children of native Swedes that were formed between 1991 and 2009. I refer to both formal marriages and cohabiting unions with common children as marriages. In the register extract used for this paper, the only available data for non-marital cohabitations with common children are that reported since 1991, which is why I have excluded marriages before that. It is a great disadvantage that it is impossible to capture unmarried cohabitors without common children because patterns of partner selection and assortative mating could be different between married and cohabiting couples (Blackwell and Lichter 2000), which could influence the results. In addition, the consequences of intermarriage for the partners potentially differ in less institutionalized types of unions (Elwert and Tegunimataka 2016). However, an advantage of using only cohabitations with common children is that children represent a certain level of involvement, similar to marriages. Moreover, the accuracy of cohabitation information in other data sources is often low (Thomson and Eriksson 2013).

The sample is restricted to birth cohorts from 1950 to 1989. The latest cohort is the last one observed in the data, and I based the choice of the earliest one on the availability of the marriage registry data (from 1968). By choosing these years, I ensure that for every individual in the data it has been possible to identify whether the marriage in the sample was a first- or higher-order marriage, both of these being included.^9^ The sample includes only native-born Swedes with two native-born parents, and I merged the partner information by using a unique identifier from the civil registration system. Couples are identified and categorized as “married” if they were either legally married or registered at the same property and had a common child. I exclude same-sex couples from the sample. I exclude marriages with second-generation immigrants from the sample as well since the focus of the paper is on immigrant–native intermarriages. I restrict the analysis to one observation per couple for the year in which the relationship was first registered (i.e. through either marriage or the birth of a common child). Natives who were not present (registered) in the country in the year prior to registration of the relationship are excluded from analysis to ensure that the union was not formed abroad. The major advantages of registry data have been the potential to include characteristics prior to marriage and avoiding that the sample is biased towards long-lasting marriages (cf. Kalmijn 1993). By assessing educational-assortative mating in the year of relationship registration, the positive effects of (inter-)marriage on education are avoided.

### Variable Description

I apply two sets of outcome variables to assess assortative mating patterns in intermarriages.^10^ The outcome variables in the first set of models are unions with immigrants from different regional origins, which are compared to the reference of native–native unions. Using a multinomial logit model, I estimate the associations between being in a union with an immigrant of a specific origin and individual characteristics of natives as well as variables describing the assortative mating pattern of the union. The main aim with this model is to compare intermarriages with immigrants from different origins (who have higher or lower status in the Swedish marriage market) to endogamous Swedish marriages. To reduce the number of outcome categories, I place partner’s region of origin into an outcome variable (*immigrant status*) of four categories. The reference category is Swedish endogamous marriages, and the three outcome categories are high status (European and other Western countries), medium status (Asian and Latin American countries), and low status (African and Middle Eastern countries). I base the categorization on previous research on the attractiveness of different origin groups in the Swedish partner market (as discussed in Sect. 3). Then, in a set of binary logit models, I restrict the sample to intermarriages alone and compare marriages between immigrants who were resident in Sweden before marriage (resident immigrants) and marriage migrants. In these models, I use a more detailed variable for the immigrants’ origin (in line with what has been discussed in the literature; Osanami Törngren 2016; Potârcă and Mills 2015), which allows a more precise account of immigrant status. Marriage migrants cannot be directly identified in the data because of the lack of information about the partners’ visa status. In line with previous studies (Niedomysl et al. 2010; Östh et al. 2011), I define marriage migrants as people who immigrate to Sweden and marry a native Swede or have a common child in the same calendar year.^11^ Most female marriage migrants in the sample who were thus identified came from Asia, particularly Thailand and the Philippines, or from Russia, Poland or South America. Male marriage migrants in the sample came in most cases from Africa, Turkey, Asia, the former Yugoslavia, and Great Britain and the USA. These countries are among the most common countries of origin for immigrants who were granted family-based immigrant visas listed in the official statistics (Statistics Sweden 2011).^12^ These regions of origin also correspond to evidence from the mail-order bride literature (Hidalgo and Bankston 2011; Constable 2012).

The major variables of interest—educational-assortative mating and age-assortative mating—capture the joint distributions of these characteristics among partners. Education is registered in seven categories consistent with the levels in the Swedish education system.^13^ Immigrant education is only registered if the education was reported in the census of 1970 or 1990, or if a degree was obtained in Sweden, or if the education was formally recognized by the Swedish higher education authorities (Högskoleverket, from 2000). Statistics Sweden has attempted to supplement the lack of information from surveys on newly arrived immigrants’ education (for 1995 and on an annual basis from 1999; Statistics Sweden 2005), but for a relatively large fraction of adult immigrants (approx. 25%) there is no information on education recorded in the year of relationship registration. The number of missing observations among intermarried immigrants is lower (18% in the year of relationship registration) but, particularly for newly arrived immigrants, the amount of missing information on education is still high since the process of formal registration and registration from the survey information is a lengthy one. To account for the delayed registration of immigrants’ education, I have replaced missing information in the year of relationship registration if the individual has an entry for education in later years. I have replaced missing information with the individual’s own educational records from any time up to 3 years later. To avoid educational attainment being an outcome of the union, I have ignored educational information that was registered four or more years after the start of a union. The time frame of 3 years ensures that those individuals studied at least started their education at the time of partnership registration. The number of missing observations is thereby reduced to 5%. The *educational-assortative mating* variable is based on the seven-category education variable and simply denotes whether the partner’s education is higher or lower than or indeed the same as the individual’s own education. Similar to educational-assortative mating, *age*-*assortative mating* is based on the partners’ age difference and has been sorted into four categories: partner older (three or more years), age homogamy (± 2 years), partner younger (3–6 years), and partner younger (seven or more years). I base this categorization on the distribution of age differences in Swedish marriages. The average age difference between native Swedish men and women is 2 years, which is why I regard marriages within this age difference category as homogamous and take them as reference.

### Methods

I have used binomial and multinomial logit models for different outcomes and have estimated the models separately by gender. The use of logit models is advantageous because these can incorporate individual characteristics (which relate to Hypothesis 2) as well as the assortative mating variables (which relate to Hypotheses 1 and 3) and give an indication of differences in marriage patterns between Swedish endogamous unions and intermarriages.^14^ In the multinomial logit model, the outcome categories are immigrant status [in terms of (1) high status, (2) medium status, and (3) low status], which is compared to native–native marriage (0). In the binary logit Models 1–3, the sample is restricted to intermarriages, and marriage with marriage migrants (1) is compared to marriage with resident immigrants (0). All models include all individual characteristics (education, income, age, relationship order, type of municipality, period, an interaction term between municipality and period), partner’s education, and partner origin (for Models 1–3). For couple characteristics, educational-assortative mating and age-assortative mating are included. In Model 2, an interaction term between partner origin and educational-assortative mating is added, and likewise in Model 3 an interaction term has been added between partner origin and age-assortative mating.

#### A Note on Opportunities

The opportunity structures of the marriage market are not addressed directly in this study. Previous research has identified group size and sex ratio to explain the intermarriage rates of different immigrant groups. However, this cannot be directly transferred to native Swedes’ marriage market opportunities, as the native group size is sufficiently large and basically sex-balanced. Moreover, studies that account for natives’ opportunities in terms of locality (Haandrikman 2014), local gender imbalance, and workplace opportunities (Östh et al. 2011) conclude that opportunity structures have relatively little impact on the propensity of natives to intermarry. Furthermore, accounting for opportunity structures in a globalized marriage market would appear to be extremely difficult. Because marriage migrants constitute a sizeable proportion of foreign spouses of native Swedes (particularly Swedish men), accounting for opportunity structures with regard to age and education in the origin countries of marriage migrants would be a necessary but challenging task.

Because immigration to and emigration from Sweden and the composition of immigrants contribute to the structure of the (inter-)marriage market, all models include interaction terms between type of municipality and period (i.e. type of municipality × period-fixed effects). These fixed effects should account for most of the aforementioned characteristics of the marriage market.

#### Limitations

This study is not without its limitations. First, it is a more descriptive account of assortative mating patterns and does not make any claims regarding causality. Second, in a comprehensive account of marriage patterns, opportunities in the marriage market should be explicitly modelled and not simply controlled for. Moreover, the marriage market controls in this study (period–region interactions) may be too rudimentary regarding both time and region to capture local marriage markets. Another impediment to this study is the rudimentary measure of marriage migration, which is likely to be inferior to, say, a direct observation of visa type. Last, demographic characteristics, which may affect status in the marriage market, are only included in the form of age and relationship order. Despite being important status characteristics, these may not be the most or indeed the only important ones. Specifically, the inclusion of measures of physical attractiveness such as body mass or height could be an important contribution when researching status exchange in immigrant–native intermarriages.

## Results

I report coefficients taken from binomial logit and multinomial logit models in their exponentiated form as odds ratios. Individual characteristics are discussed briefly, but the main focus is on educational- and age-assortative mating.

### Native Swedish Men–Immigrant Women Intermarriages

#### Comparing Immigrant–Native Intermarriages to Endogamous Swedish Marriages

In the multinomial logit model (Table 2 Panel A), both individual characteristics that are associated with intermarriage with immigrant groups of different status as well as educational-assortative and age-assortative mating variables are included. In this section, I start with discussing the coefficients of the individual characteristics, which relate to Hypothesis 2 (Attractiveness Hypothesis), and continue by discussing the assortative mating variables which relate to Hypothesis 1 (Openness Hypothesis) and Hypothesis 3 (Status Exchange Hypothesis).

**Table 2:** Estimates of multinomial logistic regression models for intermarriage of native men by immigrant status of the partner (Panel A; ref: native–native marriage) and logistic regression models for marriage migrant marriages of native men (Panel B; Model 1, ref: resident immigrant marriage)

	A				B			
	High status		Medium status		Low status		Marriage migrant	
	OR	SE	OR	SE	OR	SE	OR	SE
*Individual attractiveness*								
Education (ref: upper secondary)								
1 Primary/lower secondary	1.13	0.08	0.66***	0.07	0.92	0.17	1.16	0.14
2	1.12***	0.03	0.99	0.03	1.00	0.06	0.99	0.04
4	0.94**	0.02	0.86***	0.02	1.10	0.06	0.87***	0.03
5	0.91***	0.02	0.83***	0.02	1.24***	0.08	0.83***	0.04
6	0.93*	0.03	0.72***	0.03	1.51***	0.12	0.80***	0.05
7 Postgraduate education	1.17*	0.06	0.67***	0.06	1.95***	0.30	0.60***	0.07
Labour income (ref: lowest septile)								
2	0.84***	0.02	0.87***	0.03	0.79***	0.05	0.78***	0.04
3	0.76***	0.02	0.76***	0.02	0.72***	0.04	0.69***	0.03
4	0.72***	0.02	0.72***	0.02	0.62***	0.04	0.65***	0.03
5	0.71***	0.02	0.69***	0.02	0.56***	0.03	0.65***	0.03
6	0.71***	0.02	0.74***	0.02	0.54***	0.03	0.59***	0.03
Highest septile	0.73***	0.02	0.66***	0.02	0.51***	0.03	0.50***	0.02
Age (ref: 26–34)								
18–25	0.79***	0.02	1.06	0.03	1.11	0.07	0.81**	0.05
35–40	1.58***	0.03	1.20***	0.03	1.43***	0.06	1.23***	0.04
41 and older	2.58***	0.05	1.72***	0.04	1.97***	0.10	1.52***	0.06
Relationship order (ref: first)								
Second	1.14***	0.02	0.79***	0.02	1.06	0.05	1.14***	0.04
Third or higher	1.47***	0.06	1.04	0.05	1.59***	0.15	1.64***	0.11
Partner origin (ref: West/European)								
Nordic							0.22***	0.01
Central/East European							1.85***	0.09
Latin American							1.85***	0.10
Asian							2.59***	0.12
African							2.78***	0.19
Middle Eastern							0.60***	0.05
*Assortative mating variables*								
Educational-assortative mating (ref: homogamy)								
Hypergamy: partner higher education	1.05*	0.02	1.14***	0.03	1.39***	0.08	1.35***	0.06
Hypogamy: partner lower education	1.30***	0.03	1.62***	0.04	1.41***	0.08	1.24***	0.06
Age-assortative mating (ref: age homogamy)								
Hypergamy: partner older	1.64***	0.03	1.04	0.03	1.44***	0.08	1.07	0.05
Hypogamy: partner younger (3–6 years)	1.03	0.02	1.38***	0.03	1.45***	0.06	1.44***	0.05
Hypogamy: partner younger (7 + years)	1.32***	0.03	3.63***	0.08	3.21***	0.15	2.86***	0.10
Baseline	0.05***	0.00	0.01***	0.00	0.00***	0.00	0.11***	0.01
*N*	616,750						52,281	
Assortative mating coef. significantly different								
Hypergamy versus hypogamy	Yes		Yes		No		No	
Older versus younger (3–6 years)	Yes		Yes		No		Yes	
Older versus younger (7 + years)	Yes		Yes		Yes		Yes	

Models control for partner’s education, type of municipality of residence, and period interactions. Labour income is averaged over *t* − 4 to *t* − 1. Educational-assortative mating is based on a seven-category registration of education. Age homogamy is defined as an age gap of less than 3 years. See “Appendix” section for detailed variable labels and descriptions

*OR* odds ratios, *SE* standard errors

*p < 0.05; **p < 0.01; ***p < 0.001

^a^Wald test of equality of coefficients, significance at 5% level

Overall, the results regarding individual attractiveness show that men with characteristics that are likely to signal low attractiveness in the marriage market (lower income, being above age 40, being divorced or having children from previous relationships) are more likely to marry immigrants as predicted by the general attractiveness hypothesis. Turning to the specific variables, the coefficients show that income is negatively associated with intermarriage to immigrants in all three groups. Also, demographic characteristics that signal low attractiveness are positively associated with intermarriage. Relatively older men, particularly men aged 41 and older, have higher odds of intermarriage with immigrants in all three groups. Native Swedish men who are in higher-order relationships (both second and third and higher) show increased odds of marrying someone from either the high- *or* low-status group but men who marry someone from the medium-status group have not experienced a failed marriage more often than the reference group of endogamous Swedish men. For educational levels, however, the association between attractiveness and intermarriage is not as clear. Education is nonlinearly associated with intermarriage with high-status immigrants: men with both compulsory and higher education have higher odds of marrying an immigrant than those with intermediate education. Men married to immigrants with medium status are more likely to have had an intermediate education, and men married to immigrants with low status are more likely to have had a higher education.

These results generally show that an association of low attractiveness and intermarriage exists for men marrying immigrants, which is in line with the general attractiveness hypothesis. However, since this association exists for men who marry immigrants from both the high-status and low-status groups, the more specific formulation of the hypothesis—that men with low attractiveness in the Swedish endogamous partner market are mainly pushed towards marrying immigrants with only low status—cannot be supported. Thus, lower attractiveness in the marriage market is associated with intermarriage but not specifically that with immigrants of low status. The Attractiveness Hypothesis 2 is therefore not fully supported for men.

The results on assortative mating, in particular the results on age-assortative mating, support Hypothesis 3 (Status Exchange Hypothesis) more than Hypothesis 1 (Openness Hypothesis) as was expected for native men–immigrant women unions. The coefficients show that assortative mating by age and education differs by immigrant status of the partner. *Educational-assortative mating* patterns in native Swedish men–immigrant women intermarriages show that men who marry down in terms of education have higher odds of intermarriage with high- and medium-status immigrants and show less directed heterogamy for men who marry immigrants with low status. These estimates for educational-assortative mating do not support any interpretation in terms of the status considerations of native Swedish men. The associations between *age*-*assortative mating* and intermarriage with an immigrant of high status are fairly undirected and thus refute an interpretation of status. In contrast, age-assortative mating for men married to women of medium or low ethnic status is more pronounced: the odds of marrying someone from the low- or medium-status group are slightly increased for men in age-hypergamous unions, but in age-hypogamous relationships—particularly those with much younger partners (seven or more years)—the odds of intermarriage more than tripled in both groups. The high odds of intermarriage for age-hypogamous unions with a substantial gap are consistent with the assumption of a hierarchy of immigrants in the Swedish marriage market.

To summarize, patterns of educational-assortative mating show increased educational heterogamy and hypogamy in intermarriages compared to endogamous Swedish marriages. While this signals lower preferences for educational homogamy and could support the Openness Hypothesis (Hypothesis 1), patterns of age-assortative mating show systematic differences between endogamous marriages and intermarriages and thus refute this hypothesis. The patterns of pronounced age hypogamy among marriages with immigrants of medium or low status clearly support instead the Status Exchange Hypothesis (Hypothesis 3).

#### Comparing Two Types of Intermarriages: Resident Migrants Versus Marriage Migrants

**Fig. 2:**
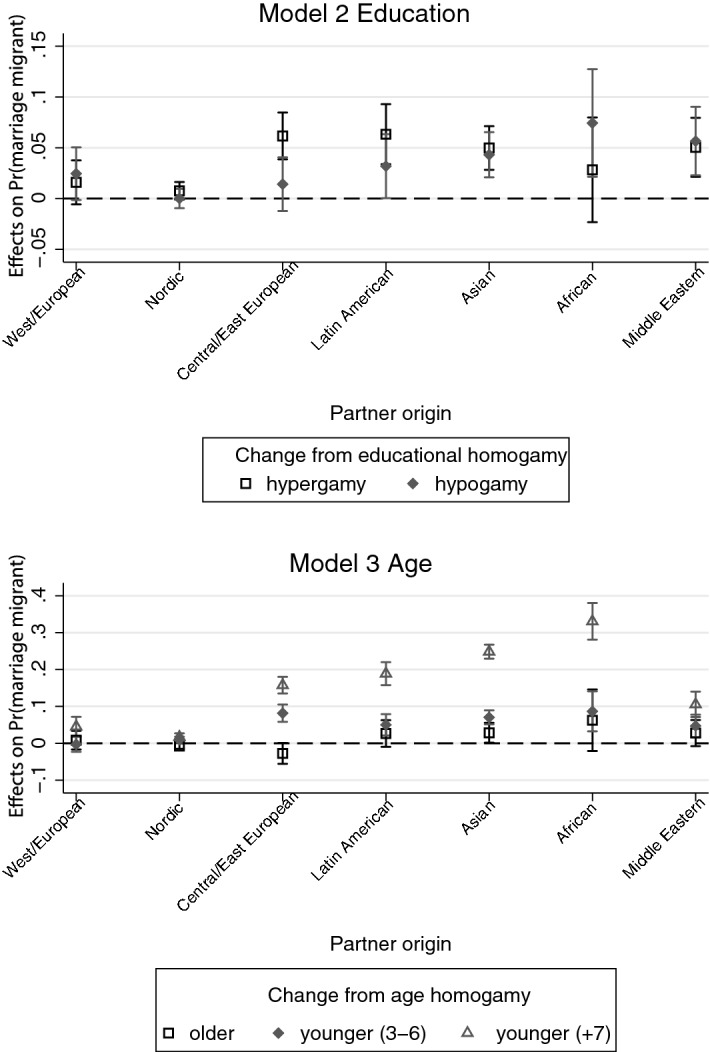
Educational- and age-assortative mating in native men’s intermarriages across partners’ origin groups. *Note*: Models control for education, partner’s education, income, age, relationship order, education-/age-assortative mating and type of municipality of residence and period interactions. Labour income is averaged over *t* − 4 to *t* − 1. Educational-assortative mating is based on a seven-category registration of education and measured in three categories: homogamy (ref.), hypergamy (partner higher education) and hypogamy (partner lower education). Age-assortative mating is measured in four categories: age homogamy (age gap of less than 3 years, ref.), partner older, partner younger (3–6 years) and partner younger (7 + years)

The binary logit model (Model 1 displayed in Table 2 Panel B, Models 2 and 3 displayed in Fig. 2) compares the odds of two intermarriage types: marriage to marriage migrants versus marriage to resident immigrants. Regarding native Swedish men’s individual attractiveness, both status in terms of income and education and status in terms of age and previous relationships are negatively associated with marrying a marriage migrant, which gives support to Hypothesis 2 (Attractiveness Hypothesis) for unions with marriage migrants. More specifically, lower levels of education and income increase the odds of marrying a marriage migrant. Older men, particularly men above age 40, and men who have experienced more than one failed relationships have increased odds of marriage to a marriage migrant (by approximately 52 and 64%, respectively). These findings suggest that men with the lowest attractiveness in the Swedish partner market are more inclined to marry marriage migrants.

In unions with marriage migrants, age-assortative mating patterns but not educational-assortative mating patterns are in line with the Status Exchange Hypothesis 3. Educational-assortative mating in unions with marriage migrants does not point to status exchange because the coefficients for educational hypergamy and hypogamy are not significantly different. Age-assortative mating, in contrast, shows a strong association with marrying a marriage migrant. Furthermore, it shows that marriages to partners from certain countries of origin are more likely to be marriages to marriage migrants than marriages to resident immigrants. This holds true for Central/East European, Latin American, and—in particular—Asian and African women, but not for Nordic or Middle Eastern women.

When interacting partner’s origin with educational-assortative mating (Model 2, Fig. 2) and age-assortative mating (Model 3, Fig. 2), the pattern of status exchange becomes more visible. The results are presented in the form of marginal effects. (Regression tables are available from the author upon request.) The origin groups are listed on the *X*-axis with the estimate for Nordic on the left and that for Middle Eastern on the right according to immigrant status (based on dating preferences; see theory section), with an exception made for the reference category (West/European is closest to the origin and will not be discussed). If immigrant status perfectly predicts status exchange patterns, I expect a continuous increase across these groups from left to right (i.e. from Nordic to Middle Eastern) for the categories (educational) “hypergamy” and “younger (7 +)”.

For education, Fig. 2 does not depict such pattern. The probability of marrying a marriage migrant is only increased for educationally hypergamous men with Central/East European and Latin American partners, but the confidence intervals of hypergamy and hypogamy overlap. However, for native Swedish men in age-hypogamous unions with a substantial gap (7 or more years), the pattern across origin groups is close to what is theoretically expected and indicates strong patterns of status exchange for the origin groups of lowest status. An exception to this pattern is unions with partners from the Middle East, which are a clear outlier. There is a continuous increase in the probability that the union is a marriage migrant union (compared to a union with a resident migrant) from Nordic to African for men with partners who are seven or more years younger, and there are no clear patterns for men with older or slightly younger partners.

### Native Swedish Women–Immigrant Men Intermarriages

#### Comparing Immigrant–Native Intermarriages to Endogamous Swedish Marriages

For native Swedish women, Hypothesis 2 (Attractiveness Hypothesis) is supported by the negative association between socioeconomic status (education and income) and intermarriage with immigrants from all three status groups (multinomial logit model, Table 3 Panel A) but less so by the associations with the demographic variables. The findings regarding the demographic characteristics, which may affect a person’s status in the marriage market, strongly resemble those for men only in marriages with immigrants of high status. Specifically, older women and women in higher-order relationships show higher odds of marrying an immigrant of high status, but it is younger women (18–25 years old) who show 41% higher odds of marrying an immigrant of medium status and 35% higher odds of marrying an immigrant of low status. Regarding previous relationships, however, women who have experienced more than one failed relationship show increased odds of intermarriage than do women in their first relationship, and this is particularly pronounced among marriages with low-status immigrants (OR of 1.48). The findings on the demographic characteristics are slightly more ambiguous and do not fully support Hypothesis 2 since being older—which should be negatively related to marriage market status, particularly for women—does not increase the odds of intermarriage, at least not in intermarriages with medium- and low-status immigrants.

**Table 3:** Estimates of multinomial logistic regression models for intermarriage of native women by immigrant status of the partner (Panel A; ref: native–native marriage) and logistic regression models for marriage migrant marriages of native women (Panel B; Model 1, ref: resident immigrant marriage)

	A				B			
	High status		Medium status		Low status		Marriage migrant	
	OR	SE	OR	SE	OR	SE	OR	SE
*Individual attractiveness*								
Education (ref: upper secondary)								
1 Primary/lower secondary	0.94	0.10	0.86	0.22	0.72	0.14	1.04	0.06
2	1.15***	0.03	1.19**	0.06	1.21***	0.05	0.90	0.04
4	0.92***	0.02	1.07	0.04	0.90***	0.03	0.76*	0.05
5	0.87***	0.02	0.93	0.05	0.73***	0.03	0.64***	0.05
6	0.81***	0.03	0.83**	0.06	0.67***	0.04	0.99***	0.24
7 Postgraduate education	0.90	0.09	0.91	0.20	0.80	0.17	2.97	0.23
Labour income (ref: lowest septile)								
2	0.81***	0.02	0.69***	0.03	0.71***	0.03	0.70***	0.03
3	0.72***	0.02	0.62***	0.03	0.60***	0.02	0.58***	0.03
4	0.65***	0.02	0.52***	0.02	0.52***	0.02	0.55***	0.03
5	0.63***	0.02	0.43***	0.02	0.43***	0.02	0.49***	0.03
6	0.63***	0.02	0.40***	0.02	0.37***	0.02	0.42***	0.03
Highest septile	0.59***	0.02	0.30***	0.01	0.26***	0.01	0.40***	0.03
Age (ref: 26–34)								
18–25	0.78***	0.02	1.41***	0.05	1.35***	0.05	1.27***	0.06
35–40	1.28***	0.03	0.82***	0.04	0.86***	0.03	0.92	0.05
41 and older	1.36***	0.03	0.67***	0.04	0.83***	0.04	1.06	0.07
Relationship order (ref: first)								
Second	0.98	0.02	0.93	0.04	0.97	0.03	1.39***	0.06
Third or higher	1.17***	0.05	1.14	0.10	1.48***	0.10	2.09***	0.19
Partner origin (ref: West/European)								
Nordic							0.17***	0.01
Central/East European							1.33***	0.07
Latin American							1.09	0.07
Asian							1.84***	0.11
African							5.99***	0.33
Middle Eastern							2.09***	0.10
*Assortative mating variables*								
Educational-assortative mating (ref: homogamy)								
Hypergamy: partner higher education	1.27***	0.03	1.41***	0.06	1.68***	0.07	1.50***	0.08
Hypogamy: partner lower education	1.12***	0.03	1.29***	0.06	1.56***	0.06	1.29***	0.07
Age-assortative mating (ref: age homogamy)								
Hypergamy: partner older	1.32***	0.02	1.04	0.03	1.17***	0.03	0.64***	0.02
Hypogamy: partner younger (3–6 years)	1.40***	0.03	2.04***	0.09	3.03***	0.12	2.09***	0.10
Hypogamy: partner younger (7 + years)	1.80***	0.07	7.44***	0.38	14.6***	0.61	5.95***	0.35
Baseline	0.06***	0.00	0.01***	0.00	0.02***	0.00	0.15***	0.01
*N*	605,474						40,373	
Assortative mating coef. significantly different								
Hypergamy versus hypogamy	Yes		No		No		No	
Older versus younger (3–6 years)	Yes		Yes		Yes		Yes	
Older versus younger (7 + years)	Yes		Yes		Yes		Yes	

Models control for partner’s education, type of municipality of residence, and period interactions. Labour income is averaged over *t* − 4 to *t* − 1. Educational-assortative mating is based on a seven-category registration of education. Age homogamy is defined as an age gap of less than 3 years. See “Appendix” section for detailed variable labels and descriptions

*OR* odds ratios, *SE* standard errors

*p < 0.05; **p < 0.01; ***p < 0.001

^a^Wald test of equality of coefficients, significance at 5% level

The patterns of educational- and age-assortative mating show more heterogamy across all intermarriage outcomes compared to endogamous Swedish marriages. As predicted by Hypothesis 1 (Openness Hypothesis), the coefficients for educational-assortative mating show generally increased heterogamy as the coefficients of educational hypergamy and hypogamy are not statistically different. This pattern points to lower general homogamy preferences and not to status exchange. For this reason, the formulation of Hypothesis 3 (Status Exchange) for women cannot be supported. Increased heterogamy could be considered to support Hypothesis 1; however, age-assortative mating in native Swedish women’s intermarriages appears to be not random but systematic. Striking patterns of age hypogamy appear for native Swedish women married to immigrants in the medium- and low-status groups: age hypogamy where the partner is seven or more years younger increases the odds of intermarriage by a factor of 7 and 15, respectively. The strong symmetry of status exchange patterns between men and women is contrary to expectations and could lead to the conclusion that women, like men, do not trade for education but for age. However, it appears counterintuitive to accept that it is younger women who have higher odds of intermarriage, contrary to the pattern found for men and that the associations between having a considerably younger partner and intermarriage are nevertheless strong in the medium- and low-status groups. When interacting women’s ages with age-assortative mating (not displayed), the patterns go in the expected direction; i.e. hypogamy patterns are particularly strong among older women (41 and older).

#### Comparing Two Types of Intermarriages: Resident Migrants Versus Marriage Migrants

A comparison of marriages with marriage migrants and marriages with resident immigrants (Table 3 Panel B) generally shows that marriage migrant marriages are associated with relatively lower status of native women in economic and demographic characteristics. An exception to this pattern is women with the highest level of education, who are as likely to marry marriage migrants as are women with intermediate education. Women with relatively lower status in characteristics such as education, income and relationship order (but not age) are more prone to marrying marriage migrants. For women who are in their third or higher committed relationship, the odds of marrying a marriage migrant are more than double compared to those in marriages with resident immigrants. These patterns support the specific formulation of Hypothesis 2 (Attractiveness Hypothesis) for unions with resident versus marriage migrants.

Assortative mating patterns in marriages with marriage migrants also display more pronounced patterns in age-assortative mating than in educational-assortative mating. Women with husbands seven or more years younger than themselves have almost six times higher odds of marrying a marriage migrant than marrying a resident immigrant. While this result is contrary to Hypothesis 3 for women, which expected that women trade for educational status, it can still be considered to support the idea of status exchange for age.

**Fig. 3:**
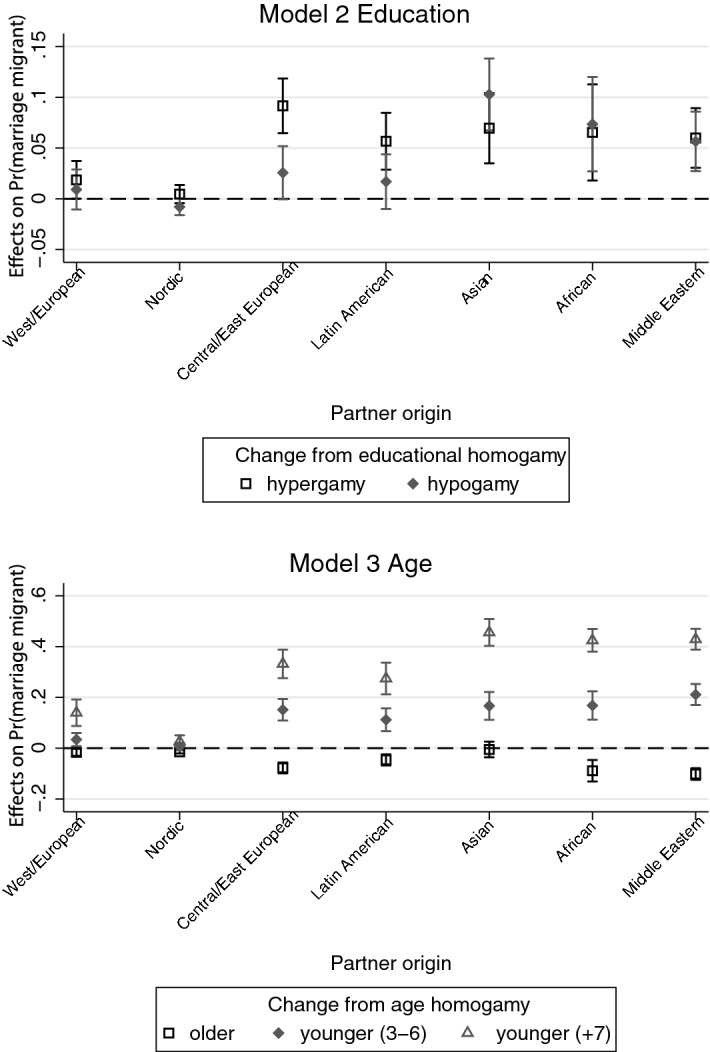
Educational- and age-assortative mating in native women’s intermarriages across partners’ origin groups. *Note:* Models control for education, partner’s education, income, age, relationship order, education-/age-assortative mating and type of municipality of residence and period interactions. Labour income is averaged over *t* − 4 to *t* − 1. Educational-assortative mating is based on a seven-category registration of education and measured in three categories: homogamy (ref.), hypergamy (partner higher education) and hypogamy (partner lower education). Age-assortative mating is measured in four categories: age homogamy (age gap of less than 3 years, ref.), partner older, partner younger (3–6 years) and partner younger (7 + years)

For women, the pattern of interacting origin group and the assortative mating variable is quite similar to that of men (Fig. 3, Models 2 and 3). The confidence intervals of educational hypergamy and hypogamy overlap for all groups except for Central/East European partners, which does not support a status exchange interpretation for most groups.

The age-assortative mating patterns, however, do support a status exchange interpretation for women. The probability of native Swedish women marrying marriage migrants is significantly different from zero in age-hypogamous unions with all non-Nordic partners (partners being 3–7 years *or* 7 or more years younger) even though the probability of marrying a marriage migrant does not increase linearly with immigrant status as was found for men.

## Conclusion

Since marriage is one of the most intimate relationships in life, marriage between different social groups has the potential to reveal the social distance between them. Theoretically speaking, if marriage means accepting one another as equals, intermarriage can be an indication of *openness* towards other social groups (Kalmijn 1991). Since marriage is also always related to status (Kalmijn 1998), adopting the openness perspective would mean that immigrant–native marriages are not expected to differ from native–native marriages with regard to status homogamy, or, if they do, this is in a random fashion. If intermarriage is related to low individual *attractiveness in the marriage market* or if there are systematic differences in *assortative mating patterns*, this can be seen as an indication that status considerations are important determinants of intermarriage. Accordingly, members of different social groups do not regard each other as equals.

This study focuses on marriages between immigrants and natives in Sweden and in particular on assortative mating patterns by education and age. Marriage market status both in terms of economic and demographic characteristics of native Swedes is associated with intermarriage. The findings show that—broadly speaking—native Swedish men and women with lower status in economic and demographic characteristics are more prone to intermarry, which is similar to the findings of Östh (2011) and Haandrikman (2014). While these patterns support the idea that individuals of lower status in the marriage market suffer from a competitive disadvantage and are hence more likely to marry immigrants (Fu 2001; Gullickson and Torche 2014), the patterns found among native men also support the idea that the highly educated are more likely to be open towards immigrants (Hello et al. 2002; Wagner and Zick 1995).

Moreover, the findings of this study show that there are systematic differences in assortative mating patterns between native endogamous marriages and intermarriages, which would support a status exchange interpretation. With respect to educational-assortative mating, intermarriages display higher levels of educational heterogamy across immigrant groups with different levels of status in society, but the patterns appear to be more random than systematic. With respect to age, however, age-assortative mating patterns display systematic differences that indicate the existence of *age status exchange* in Swedish intermarriages. Intermarriages are generally more heterogamous with regard to age as well. The patterns of age hypogamy with a substantial gap for native Swedish men *and* women along the lines of a hierarchy of immigrants nonetheless indicate that for some immigrant groups intermarriage with natives is more achievable when they have other attractive characteristics to offer in return. These associations are particularly pronounced for intermarriages with marriage migrants, where these patterns closely follow the theoretically predicted hierarchy of immigrants. Marriage migrants can be regarded as a different category to resident immigrants because the union with a Swede gives them the opportunity to be given secured residence, which is likely to make them more receptive to status exchange.

Sweden often appears to be a comparatively open society with low levels of educational homogamy and high levels of gender equality. Similarly, intermarriage between immigrants and natives has been thought of as indicating the openness of natives towards immigrants and accepting them as “equal lifetime partner[s]” (Kalmijn 1991). The findings of this study challenge this view and support the notion that country of birth serves as a boundary in the native marriage market. This study illustrates the fact that this boundary manifests itself not only by excluding immigrants of certain immigrant groups from the pool of marriage partners but also by allowing them in if they have something to offer in return. In the Swedish case, this is likely to be age.

## Appendix

See Table 4.

**Table 4:** Sample and variable description for native Swedish men and women included in the sample

Variable	Description and values	Men		Women	
		*N*	Per cent	*N*	Per cent
Intermarriage—hierarchy of immigrants	Outcome variable multinomial logit model	564,469	91.52	565,101	93.33
	*Native marriages*				
	Intermarriage				
	*High status*	27,776	4.50	25,724	4.25
	*Medium status*	20,417	3.31	6723	1.11
	*Low status*	4088	0.66	7926	1.31
Intermarriage type	Outcome variable binary logit models				
	Resident immigrant	41,787	79.93	33,943	84.07
	Marriage migrant	10,494	20.07	6430	15.93
Age at marriage	18–25	358,425	0.58	342,300	0.57
	26–34	79,424	0.13	138,163	0.23
	35–40	108,754	0.18	77,714	0.13
	41 and older	70,147	0.11	47,297	0.08
Education	1 Primary/lower secondary education less than 9 years	3080	0.50	1381	0.23
	2 Primary and secondary education 9–10 years	69,948	11.34	51,762	8.55
	3 Upper secondary education 2 years or less	209,528	33.97	163,466	27.00
	4 Upper secondary education 3 years	134,615	21.83	153,599	25.37
	5 Post-secondary education less than 3 years	91,279	14.80	91,353	15.09
	6 Post-secondary education 3 years or more	103,466	16.78	141,674	23.40
	7 Postgraduate education	4834	0.78	2239	0.37
Partner’s Education	1 Primary/lower secondary education less than 9 years	5108	0.83	7543	1.25
	2 Primary and secondary education 9–10 years	55,936	9.07	67,889	11.21
	3 Upper secondary education 2 years or less	157,321	25.51	200,647	33.14
	4 Upper secondary education 3 years	154,070	24.98	129,799	21.44
	5 Post-secondary education less than 3 years	95,549	15.49	91,159	15.06
	6 Post-secondary education 3 years or more	146,131	23.69	103,384	17.07
	7 Postgraduate education	2635	0.43	5053	0.83
Income	CPI-adjusted annual income from labour, averaged over 4 years prior to marriage. Incomes below one price basic amount are excluded. The price basic amount is used for calculating different kinds of benefits in the social insurance system; incomes below this amount are very low and not self-sufficient. Including low and zero incomes does not change results				
	Lowest septile	88,106	14.29	86,495	14.29
	2	88,103	14.29	86,497	14.29
	3	88,109	14.29	86,492	14.29
	4	88,107	14.29	86,499	14.29
	5	88,110	14.29	86,498	14.29
	6	88,107	14.29	86,494	14.29
	Highest septile	88,108	14.29	86,499	14.29
Relationship order	Based on partners’ civil registration in dwelling units. Included are cohabiting married couples and unmarried cohabiting couples with common children. Cohabiting couples without common children cannot be identified in the Swedish civil registration system in the period under study. Because information on cohabitation with common children is only available from 1990 onwards, cohabitations with common children that were first registered before 1990 are not included in the variable and the number of lower order relationships may be overstated				
	First	526,324	85.34	505,767	83.53
	Second	83,063	13.47	90,034	14.87
	Third or higher	7363	1.19	9673	1.60
Partner origin	Based on partner’s country of birth. For countries of origin with less than 100 persons and for relationships not registered until after 2005, only a broader grouping of countries is available and used for categorization. Where grouping into one of the origin categories was impossible, observations were excluded from all models (less than one per cent of observations, mainly immigrants from Europe)				
	Nordic	6138	11.74	9457	23.42
	West/European	13,481	25.79	11,879	29.42
	Central/East European	8157	15.60	4388	10.87
	Latin American	5018	9.60	3839	9.51
	Asian	15,399	29.45	2884	7.14
	African	1818	3.48	2820	6.98
	Middle Eastern	2270	4.34	5106	12.65
Municipality of residence	Classification of Swedish municipalities by the Swedish Association of Local Authorities and Regions. The classification is based on structural characteristics such as population, commuting patterns, tourism and travel industry and economic structure				
	Metropolitan municipalities	107,088	17.36	108,219	17.87
	Suburban municipalities	88,091	14.28	87,324	14.42
	Large cities	191,739	31.09	187,683	31.00
	Suburban municipalities to large cities	21,246	3.44	20,547	3.39
	Commuter municipalities	45,351	7.35	43,876	7.25
	Tourism and travel industry municipalities	19,082	3.09	18,799	3.10
	Manufacturing municipalities	52,591	8.53	50,225	8.30
	Sparsely populated municipalities	11,326	1.84	10,923	1.80
	Municipalities in densely populated regions	56,709	9.19	55,051	9.09
	Municipalities in sparsely populated regions	23,527	3.81	22,827	3.77
Period	1991–1995	160,876	26.08	163,261	26.96
	1996–2000	143,678	23.30	140,813	23.26
	2001–2005	168,953	27.39	163,765	27.05
	2006–2009	143,243	23.23	137,635	22.73

## Footnotes

1 In the literature, the term intermarriage is often used to describe marriages across social groups such as intermarriage between educational groups. I use it exclusively to refer to immigrant–native marriages.

2 In the context of Sweden, few studies have focused on natives (Haandrikman 2014; Niedomysl et al. 2010; Östh et al. 2011), and they are either more descriptive by nature or analyses of individual characteristics that are associated with intermarriage rather than joint characteristics of couples.

3 The use of the terms hypergamy and hypogamy in relation to educational and age homogamy can be confusing. Since hypergamy denotes “marrying up”, I use the term to refer on a gender-neutral basis to marrying someone who either has a higher education or is older. Likewise, hypogamy relates to marrying someone who either has a lower education or is younger. It should, however, be remembered that this corresponds to marrying up in status only with regard to education and not with regard to age since younger age denotes higher status in the marriage market.

4 With the term native, I refer to native-born men and women who have two Swedish-born parents, not to all native-born individuals.

5 If some minority groups are completely excluded from the natives’ marriage market, this would be a strong indication of closure. For completely excluded groups, closure would be reflected in intermarriage rates but not in assortative mating patterns. Since the focus of this study is on the characteristics of immigrant-native unions, openness here refers only to assortative mating patterns.

6 Gullickson and Torche (2014) describe the notion of an individual’s desirability in the marriage market as *market exchange.*

7 Because educational homogamy is dominant even in interracial marriages, most authors expect patterns of hypogamy and hypergamy to differ *relative* to intra-racial unions (see, for example, Gullickson 2006).

8 I do not regard age as the only important non-economic characteristic, which is related to status in the marriage market, but it is the only available measure in the data since this does not contain information on body mass, height, or other attractiveness measures for the general population.

9 This means that individuals who married more than once in the period 1991–2009 have been included in the sample more than once, and this includes approximately ten per cent of observations (for both men and women).

10 An entire list of covariates, including variable descriptions, can be found in “Appendix” section.

11 This definition of a marriage migrant is rather conservative but encompasses those migrants who lived with their partners after arrival. The information on couples comes from a stock register that contains data on the registered population as per 31 December of the year in question, and where immigration was registered between January and December of the same year. In the case of delayed registration of migration, this definition might be too narrow. I therefore re-estimated the models using a wider definition of marriage migrants (i.e., immigrating to Sweden and being registered with a partner in the same or the next calendar year), which does not impact greatly on the results.

12 The number of marriage migrants identified in the data accounts for approximately 20–50% of all (adult) immigrants who were granted family-related residence permits for the most common marriage migrant countries, not all of whom would appear to have migrated to marry Swedish partners. A large proportion of marriage migrants migrates to marry second-generation immigrants in Sweden.

13 This categorical variable does not allow as fine a distinction as a continuous measure of education would, and hence poses the problem that differences in the educational categories of partners could be qualitatively different with respect to different educational levels. A continuous measure of years in education is not, however, available in the data.

14 A common concern with the models applied is that they do not account for the marginal distribution of education among immigrant groups in the way the more frequently used log-linear models do. As demonstrated by Hou and Myles (2013), logit models have the same properties as log-linear models in their ability to adjust for marginal distributions and, when correctly specified, they yield the same coefficients for the exchange parameters and goodness of fit. Logit models do have the advantage as well of simplifying the model specification and facilitating parameter interpretation. Moreover, logit models can include additional covariates such as socioeconomic status measures and other variables that capture attractiveness in the marriage market. A table comparing the estimates of log-linear models with the estimates obtained by different logit models is available from the author upon request.
